# Longitudinal Changes in Mitochondrial DNA Copy Number and Telomere Length in Patients with Parkinson’s Disease

**DOI:** 10.3390/genes14101913

**Published:** 2023-10-07

**Authors:** Alberto Ortega-Vázquez, Salvador Sánchez-Badajos, Miguel Ángel Ramírez-García, Diana Alvarez-Luquín, Marisol López-López, Laura Virginia Adalid-Peralta, Nancy Monroy-Jaramillo

**Affiliations:** 1 Departamento de Sistemas Biológicos, Universidad Autónoma Metropolitana, Unidad Xochimilco, Mexico City 04960, Mexico; aortega@correo.xoc.uam.mx (A.O.-V.); mlopez@correo.xoc.uam.mx (M.L.-L.); 2 Doctorado en Ciencias Biológicas y de la Salud, Universidad Autónoma Metropolitana, Unidad Xochimilco, Mexico City 04960, Mexico; salsb0889@gmail.com; 3 Departamento de Genética, Instituto Nacional de Neurología y Neurocirugía, Mexico City 14269, Mexico; dr.miguelangelrg@gmail.com; 4 Laboratorio de Reprogramación Celular del Instituto de Fisiología Celular UNAM en el Instituto Nacional de Neurología y Neurocirugía, Mexico City 14269, Mexico; danliuc@hotmail.com (D.A.-L.); adalid.laura@yahoo.com (L.V.A.-P.)

**Keywords:** Parkinson’s disease, telomere length, mitochondrial DNA copy number, dopaminergic replacement therapy, aging, inflammatory index, IL-17A

## Abstract

Parkinson’s disease (PD) pathophysiology includes mitochondrial dysfunction, neuroinflammation, and aging as its biggest risk factors. Mitochondrial DNA copy number (mtDNA-CN) and telomere length (TL) are biological aging markers with inconclusive results regarding their association with PD. A case–control study was used to measure TL and mtDNA-CN using qPCR in PBMCs. PD patients were naive at baseline (T0) and followed-up at one (T1) and two (T2) years after the dopaminergic treatment (DRT). Plasmatic cytokines were determined by ELISA in all participants, along with clinical parameters of patients at T0. While TL was shorter in patients vs. controls at all time points evaluated (*p* < 0.01), mtDNA-CN showed no differences. An increase in mtDNA-CN and TL was observed in treated patients vs. naive ones (*p* < 0.001). Our statistical model analyzed both aging markers with covariates, showing a strong correlation between them (r = 0.57, *p* < 0.01), and IL-17A levels positively correlating with mtDNA-CN only in untreated patients (r = 0.45, *p* < 0.05). TL and mtDNA-CN could be useful markers for monitoring inflammation progression or treatment response in PD. DRT might modulate TL and mtDNA-CN, reflecting a compensatory mechanism to counteract mitochondrial dysfunction in PD, but this needs further investigation.

## 1. Introduction

Parkinson’s disease (PD) is the second most common neurodegenerative disorder, characterized as an age-related progressive disorder with an increased global prevalence of 21.7% from 1990 to 2016 [1,2]. The cardinal motor symptoms of PD are caused by the loss of dopaminergic neurons in the substantia nigra [3].

Some of the pathways implicated in the pathophysiology of PD include mitochondrial dysfunction [4] and neuroinflammation [5]. These pathways are interrelated and, along with the erosion of the telomeres, are contributing factors to the aging process [6,7]. At the same time, all these factors influence the onset and progression of this movement disorder [5]. Comprehending the participation of aging in PD could be useful because this is a proven, robust, and non-modifiable risk factor [8].

It is also relevant to evaluate the inflammatory process in PD. Particularly, cytokines as inflammatory mediators have been proposed as biomarkers [9,10,11]. Interestingly, cytokine IL-17 has been linked to progression in PD patients and neurodegeneration in mouse models [12,13]. On the other hand, some studies found that the neutrophil lymphocyte ratio or index (NLI), the Systemic Immune-inflammation Index (SII), and similar ratios can be used as potential markers of systemic inflammation [14,15]. NLI has been reported as closely related to PD [16].

Telomeres (T) are repetitive sequences (TTAGGG)n that protect the end of the chromosomes, and their length (L) shortens with each cell division; thus, TL is considered an aging marker [17]. Shorter telomeres have been associated with mortality and increased rates of age-related diseases, including Alzheimer’s disease and PD [18].

On the other hand, there is growing evidence that mitochondrial dysfunction is a relevant player in the development of PD, including the inhibition of mitochondrial complexes I and II and its implication in the epidemiology of sporadic PD [19]. Somatic mtDNA deletions of various sizes have been described at the single substantia nigra neuron level in PD [20], which in turn may disrupt critical processes in the function of neurons (e.g., ATP production, calcium buffering, oxidative stress) [21,22].

The number of mitochondrial genomes in a cell and the degree of heteroplasmy may indicate mitochondrial allostatic load, showing a large amount of variation within cells, tissues, and individuals [23]. In recent years, changes in mtDNA copy number (mtDNA-CN) have been related to mental disorders and symptom severity of various diseases, including autism, neurodegenerative diseases, cancer, and traumatic brain injury [24,25,26,27,28]. The relative quantification of mtDNA-CN has been shown to positively correlate with oxidative stress [29], energy reserves, and mitochondrial membrane potential [30]. Therefore, altered levels of this marker have been proposed as a proxy of mitochondrial dysfunction (a hallmark of aging), and its alteration reflects mitochondrial biogenesis and function [31]. Data regarding these two aging markers (TL and mtDNA-CN) and their association with PD are inconclusive and have been reported almost exclusively in Caucasian and Asian PD patients [22,28,32,33]; nevertheless, these markers vary according to ethnicity [34,35].

Dopaminergic replacement therapy (DRT) is a standard and effective treatment widely used to significantly improve motor symptoms in patients with PD. DRT mainly includes dopamine agonists (pramipexole), a dopamine metabolism precursor (levodopa), or a combined treatment (a dopamine agonist combined with a dopamine precursor or other drugs). As far as we know, only three studies have addressed the relationship between the aforementioned markers of aging and DRT in patients with PD [17,21,22].

Current challenges in PD include the difficulty in establishing a definitive diagnosis in the early stages and the predictions of disease progression. Therefore, there is a need to identify biomarkers with diagnostic and prognostic value for patients with PD. In this regard, we aimed to longitudinally assess mtDNA-CN and TL in peripheral blood mononuclear cells (PBMCs) from sporadic PD patients and healthy controls (CT), together with plasma levels of cytokines at baseline, to determine whether these measures could act as a biomarker for PD. The patient’s group exhibited shortened TL and similar mtDNA-CN compared to CT, and within the patients’ group, mtDNA-CN and TL were higher in those treated than the untreated. Our preliminary results suggest that erosion of TL and the changes of mtDNA-CN might be related to inflammation particularly associated with IL-17A plasma levels, and dopamine agonists/precursors treatment might modulate TL and mtDNA-CN in PD patients. However, future studies should confirm these findings.

## 2. Materials and Methods

### 2.1. Study Design and Participants

Untreated patients with a clinical diagnosis of PD who are seen at the Movement Disorders Clinic of our institution (Instituto Nacional de Neurología y Neurocirugía Manuel Velasco Suárez, INNNMVS) were invited to participate in this longitudinal study for two years. Some of the patient information was previously published [36]. For the present study, peripheral blood mononuclear cells (PBMCs) from 27 naive PD patients and 22 CT at baseline (T0) were available. PD patients were sampled at one (T1) and two years (T2) after the start of the dopaminergic replacement therapy (DRT). PBMC samples from patients with PD and healthy CT were matched for age, sex, BMI, and ethnicity. However, some participants of both groups were lost in the following years; therefore, the number of DNA samples was as follows: 21 patients and 18 CT for TL and mtDNA-CN in T1; 8 patients and 10 CT for TL; and 9 patients and 12 CT for mtDNA-CN analysis in T2.

This study was carried out in accordance with the latest version of the Declaration of Helsinki, and the study design was reviewed and approved by the local ethical and research committees. Written informed consent was obtained from all participants after the nature of the procedures had been fully explained (approval code INNN_38/19).

### 2.2. Assessment of Relative Quantification of the Ratio of Telomere Length and mtDNA Copy Number

Genomic DNA from PBMCs of PD patients and CT was isolated using the salting-out technique [37], followed by the assessment of telomere length (TL) and mitochondrial DNA copy number (mtDNA-CN) via real-time quantitative PCR (qPCR) using a relative quantification method, as previously described [38,39]. The telomeric (T) sequence was amplified, and a single-copy (S) control gene (*b-globin*) was used for normalization. Telomeric primer sequences used were as follows: 5′-GGTTTTTGAGGGTGAGGGTGAGGGTGAGGGTGAGGGT-3′ (forward) 5′-TCCCGACTATCCCTATCCCTATCCCTATCCCTATCCCTA-3′ (reverse); and 5′-GCTTCTGACACAACTGTGTTCACTAGC-3′ and 5′-CACCAACTTCATCCACGTTCACC-3′ for the reference gene. For the mtDNA-CN levels, the mitochondrial *ND3* gene and the nuclear (n) *TH* gene as reference, were amplified. Primer sequences used were as follows: 5′-ACACCCTCCTAGCCTTATAC-3 and GATATAGGGTCGAAGCCGC-3′ for mt-*ND3*; 5′-AGGGTATCTGGGCTCTGG-3′ and 5′-GGCTGAAAAGCTCCCGATTAT-3′ for n*TH* gene. For both quantifications, a standard curve of serial dilutions of a control DNA sample was included in each run (commercial DNA from CEPH individual 1347-02) (Thermofisher, Écublens, Switzerland). A relative measure of TL and mtDNA-CN was calculated as a T/S ratio and as the mtDNA content relative to nDNA, respectively. All PCRs were performed on the QuantStudio™ 5 Flex system (Thermofisher, Écublens, Switzerland) using the SYBR^®^ green PCR master mix kit (Applied Biosystems, Sparta, NJ, USA). All DNA samples were run in four replicates on separate plates but in the same well positions.

### 2.3. Cytokines and Blood Count

The cytokines levels were determined via enzyme-linked immunosorbent assay (ELISA) in plasma samples from patients and controls at T0 to explore the overall effect of an inflammatory response. Briefly, an Elabscience kit (Wuhan, China) was used for IL-35 determination, following the manufacturer’s instructions. Invitrogen kits were used to determine the levels of the granulocyte-macrophage colony-stimulating factor (GM-CSF), IFN-γ, IL-1b, IL-4, IL-6, IL-10, IL-12p70, IL-13, IL-17α, the transforming growth factor beta (TGF-β), and TNF-α following the manufacturer’s protocols. TGF-β was measured using the TGF-β1 ELISA Ready-Set-Go, which includes an acid treatment to detect both the mature cytokine and the TGF-β1 latency-associated peptide (LAP). The detection limits were 2 pg/mL for IL-1β, IL-4, IL-6, and IL-10; 4 pg/mL for IL-13, IFN-γ, TNF-α, IL-12p70, and IL-17α; 6 pg/mL for GM-CSF; 8 pg/mL for TGF-β; and 9.38 pg/mL for IL-35 [36]. A hemogram was also included for the total cell blood count for patients and healthy CT.

### 2.4. Clinical Data Evaluated in Patients

At baseline, the clinical data of PD patients were evaluated by at least two movement disorder specialists, including Hoen and Yahr scale (HY), the Movement Disorders Society-sponsored version of the Unified Parkinson’s Disease Rating Scale (MDS-UPDRS) total scale, Schwab and England Activities of Daily Living (SE-ADL) scale, and Beck’s Depression Inventory (BDI).

### 2.5. Statistical Analyses

All statistical analysis was performed using the R Statistical Software (version 4.3.1; R Foundation for Statistical Computing, Vienna, Austria). Data for categorical variables are presented as numbers and frequencies and as mean values with standard deviation and ranges for continuous variables, according to the normality of the Shapiro–Wilk test. Statistical significance was set at *p* < 0.05. The normality test revealed that TL and mtDNA-CN in patients and mtDNA-CN in controls were nonparametric data, whereas TL data in controls were parametric. Therefore, the mean data of both study groups were compared by the two-sample *t*-test assuming unequal variances, Kruskal–Wallis test, and the analysis of variance (ANOVA) test. The comparison analyses between mtDNA-CN and TL with demographic data and diverse variables (treatment status, immunologic status, and clinical scales) were evaluated using the Kruskal–Wallis test and ANOVA test. Boxplots were used to compare mtDNA-CN and TL between patients and controls. A multiple linear regression analysis of variance was performed with all covariables, and a multiple correlation analysis was performed to determine the association between mtDNA-CN and TL with sex, age, BMI, and immunologic status as variables. The results were presented as follows: heatmaps were used to show the correlation in controls and patients with PD and the multiple correlation analysis between TL, mtDNA-CN, clinical scales for depression (BDI), and the staging of the functional disability of patients (SE-ADL). In addition, a multiple linear regression model was performed to show the Pearson correlation between mtDNA-CN and TL with BMI, NLI, SII, TNF-a, TGF-b, IL-17a, HY, SE-ADL, total UPDRS and the DRT used. Then, a heatmap was used to depict the multiple Pearson correlations between aging markers and variables with significant values.

## 3. Results

### 3.1. Demographic and Clinical Characteristics of Participants

The study sample at T0 comprised 27 patients with PD, of which 56% were males (*n* = 15), and 22 controls (55% males, *n* = 12). Most of the patients received dopamine agonists as treatment (70.4%), followed by a combination of an agonist plus a precursor of dopamine (18.5%) and dopamine precursors (11.1%). The mean age was 61.17 ± 10.68 and 55.73 ± 10.20 years for patients and controls, respectively (Table 1). Patients and controls were paired by sex, age, and BMI (all *p*-values were >0.05). Regarding immunological parameters at T0, only the levels of IL-17A showed a significant difference between patients and controls (*p* = 0.016) (Table S1). The NLI and SII ratios were found to be similar when comparing patients vs. controls; *p* = 0.2901 and *p* = 0.2825, respectively (Table S1). These results suggest that none of the individuals included in this study showed signs of inflammation at least at T0.

In PD patients at T0, the median of the HY scale was 2 (range: 1–4), whereas the mean value of the MDS-UPDRS total score was 47.53 ± 22.38. The SE-ADL and the BDI scales showed median values of 90 (range: 20–100) and 10 (range: 0–30), respectively. Demographics for both groups, immunological parameters, and the rest of the clinical scales evaluated at baseline are shown in Table 1.

### 3.2. Longitudinal Comparison of mtDNA Copy Number in Patients vs. Controls

**Figure 1:**
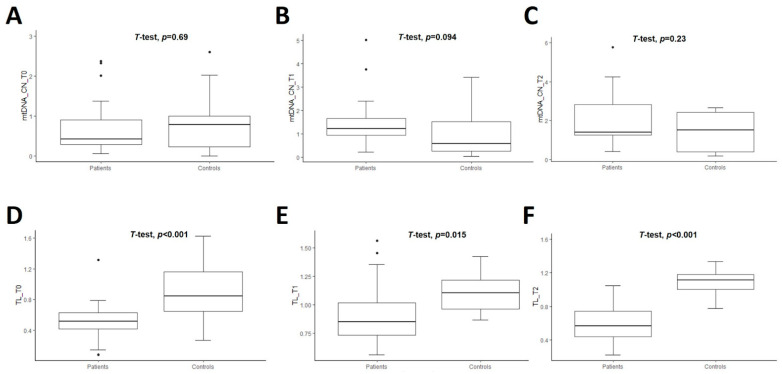
Boxplot of comparison of mtDNA copy number (mtDNA-CN) (**A**–**C**) and telomere length (TL) (**D**–**F**) in patients (*n* = 27) and controls (*n* = 22) at three time-points. T0 corresponds at baseline when PD patients were naive. T1 and T2 mean the follow-up of the patients at one and two years, respectively, after initiating the dopaminergic treatment. Student’s *t*-test was used and *p*-values are shown for each comparison.

**Figure 2:**
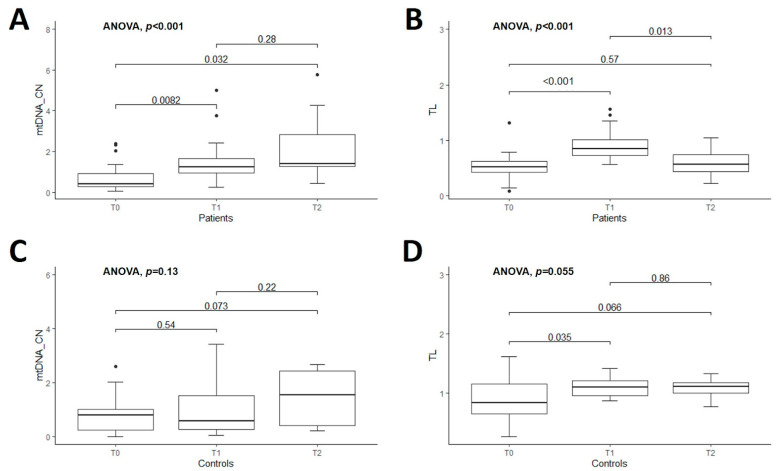
Boxplot of comparison of mtDNA copy number (mtDNA-CN) and telomere length (TL) among patients with Parkinson’s disease (*n* = 27) at three assessment time-points (T0-T1-T2) are shown in the upper figures: (**A**,**B**), respectively. Boxplot of comparison of mtDNA-CN and TL among controls (*n* = 22) at three assessed time-points (T0-T1-T2) are shown in the lower figures (**C**,**D**), respectively. T0 corresponds at baseline when PD patients were naive. T1 and T2 indicate the follow-up of the patients at one and two years, respectively, after initiating the dopaminergic treatment. ANOVA test was used for comparisons. In each box, bars with the corresponding *p*-values are indicated.

The mean value of the relative quantification of mtDNA-CN of untreated patients vs. controls was similar at T0 (0.71 ± 0.65 vs. 0.79 ± 0.68, *p* = 0.69). The comparisons of mtDNA-CN in treated patients vs. CT were also nonsignificant at T1 (1.52 ± 1.14 vs. 0.95 ± 0.91, *p* = 0.09) and at T2 (2.24 ± 1.76 vs. 1.40 ± 1.00, *p* = 0.23) (Figure 1A–C) (Table S2). However, there was a significant difference between the mean value of mtDNA-CN in untreated patients vs. treated patients for one or two years of DRT (i.e., T0 vs. T1, and T0 vs. T2 in PD patients): 0.71 ± 0.65 vs. 1.52 ± 1.14, *p* < 0.01 and 0.71 ± 0.65 vs. 2.24 ± 1.76, *p* < 0.01, respectively (Figure 2A). However, the comparison of this marker within controls was nonsignificant (T0 vs. T1, *p* = 0.53 and T0 vs. T2, *p* = 0.07) (Figure 2C) (Table S2).

### 3.3. Longitudinal Comparison of Telomere Length in Patients vs. Controls

The mean TL was significantly shorter in PD patients than CT at all time points studied, as shown in Figure 1D–F. The comparisons of TL between PD patients and controls were 0.53 ± 0.24 vs. 0.90 ± 0.36, *p* < 0.01 at T0; 0.91 ± 0.28 vs. 1.10 ± 0.17, *p* = 0.02 at T1, and 0.59 ± 0.27 vs. 1.09 ± 0.18, *p* < 0.01 at T2 (Table S2). Then, the comparison of this marker within patients was significant at T0 vs. T1 (*p* < 0.01) and T1 vs. T2 (*p* = 0.01) (Figure 2B and Table S2). The mean value of TL within controls was marginally significant only when comparing T0 vs. T1, *p* = 0.04. The remaining comparisons for CT were nonsignificant (Figure 2D and Table S2).

### 3.4. Analysis of Both Aging Markers in Untreated and Treated PD Patients with Dopaminergic Therapies

The significant longitudinal differences of both markers of aging, exclusively in the group of patients with PD, prompted us to evaluate them by subgrouping each DRT: dopamine agonists (pramipexole), dopamine precursors (levodopa), and co-treatment (levodopa + pramipexole), and only T0 vs. T1 findings were compared. The comparisons of T0 vs. T1 revealed significant differences for mtDNA-CN in the co-treatment group and for TL in the pramipexole group and co-treatment group (*p* ≤ 0.01, Kruskal–Wallis and ANOVA test) (Table S3). The comparison with T2 was not considered in this analysis due to the few patients present (Table S2).

### 3.5. Multiple Correlation Analysis of the Two Aging Markers in Both Groups of Study at Baseline

**Figure 3:**
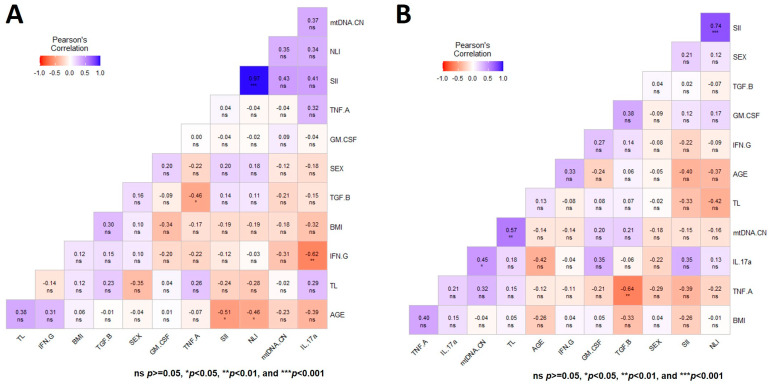
Heatmap depicting the multiple Pearson correlation between TL, mtDNA-CN, sex, age, BMI, and immunologic status variables. (**A**) Heatmap depicting the correlation in controls. (**B**) Heatmap depicting the correlation in patients with Parkinson’s disease. Significance is denoted by asterisks as follows: * *p* < 0.05, ** *p* < 0.001, and *** *p* < 0.0001.

**Figure 4:**
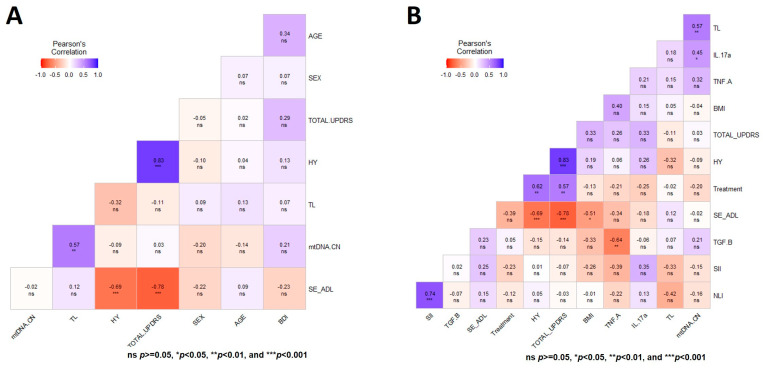
Heatmap matrix depicting the Pearson multiple correlation analysis in patients with Parkinson’s disease. (**A**) Correlation analysis between TL, mtDNA-CN, clinical scales for the staging of the functional disability of patients, and depression in patients. (**B**) Heatmap depicting the multiple correlation between TL, mtDNA-CN, and variables with significant value. Significance is denoted by asterisks as follows: * *p* < 0.05, ** *p* < 0.001, and *** *p* < 0.0001.

A general multivariate regression model was used to depict the Pearson correlation matrix between TL, mtDNA-CN, and variables such as sex, age, BMI, SII, NLI, and immunologic status, showing no significant correlation in CT for both aging markers at T0 (Figure 3A). In contrast, the heatmap displaying the same multiple correlation statistics at baseline in untreated PD patients showed that TL and mtDNA-CN were positively correlated (r = 0.57, *p* < 0.001) (Figure 3B). This correlation persisted even after adjusting for diverse covariables (Figure 4A,B). In CT, this correlation was negative and did not show significance (Figure 3A). Of note, peripheral mtDNA-CN correlated directly with IL-17A levels in untreated PD patients (T0, r = 0.45, *p* < 0.05) (Figure 3B and Figure 4B).

No significant Pearson correlations were observed between both aging markers with the HY, UPDRS, SE-ADL, or BDI scales in untreated PD patients (Figure 4A). Figure 4B shows the heatmap matrix with the correlation analysis between TL, mtDNA-CN, and only the variables with significant values. These analyses show no significant correlations of mtDNA-CN and TL in untreated PD patients with any of the studied clinical parameters (Figure 3 and Figure 4).

## 4. Discussion

Herein, we evaluated two aging markers, immunological status, clinical parameters, and inflammatory indexes in healthy CT and naive patients with sporadic PD who were followed up and sampled one and two years after the initiation of dopamine replacement therapy.

At baseline (T0), the PD patients were naive to the treatment and showed bilateral involvement without impairment of balance as rated using the HY scale. In addition, according to the categorized severity of the MDS-UPDRS scale [40], the sample showed mild symptoms on all subscales (parts I-III), and there were no reported motor complications. The severity of the motor examination (the MDS-UPDRS part III) in our sample was similar to another Mexican report [41]; however, it differs from the regions of first and third-world countries, where patients are diagnosed early and belatedly [42,43]. Furthermore, the depressive symptoms in PD patients by the BDI were minimal. Regarding the SE-ADL scale, the patients were qualified as “completely independent” and able to perform all tasks with some degree of slowness, difficulty, or disturbance.

Herein, PD patients exhibited shortened TL and similar mtDNA-CN compared to CT. In relation to TL, some studies have shown that somatic cells with a higher number of mitotic divisions exhibit a telomere shortening. In PD patients, this shortening may be accelerated by various factors such as stress, producing telomere fragility in various cells including circulating leukocytes [44,45] and the buccal epithelium [46]. However, a meta-analysis found no evidence of telomere shortening in patients with PD [33]. On the contrary, other investigations have found telomere lengthening in patients with PD [47,48], and several studies did not observe differences in TL in leukocytes [49,50] and in the pars compacta of the substantia nigra (SNc) [48] between PD patients and controls.

Albeit the Mendelian randomization approach that was used to examine the association between TL and PD using GWAS summary statistics in a large sample of PD patients and controls, the authors did not find a causal relationship between TL and PD [51]. Due to the inconsistency of the results, the possibility of using TL as a marker for PD is not entirely clear [52]. However, these studies did not evaluate the correlation of TL with cognition, medical treatment, or other variables. Only one paper reports that the presence of long telomeres at the time of diagnosis might be a risk factor for progression to dementia in idiopathic PD [50].

Regarding mtDNA-CN in PD, some studies have shown that PD patients have lower mtDNA-CN in blood compared to healthy CT [28,53,54]; as well as unchanged mtDNA-CN in patients compared to CT [55,56]. In a manner similar to our results, a recent study has documented an increased blood-derived mtDNA-CN in African ancestry PD patients, using ddQPCR, a more sensitive technique [32].

A strong and significant correlation between both markers of aging was observed in untreated PD patients at T0 (r = 0.57, *p* < 0.001); however, in T1 and T2, there was statistical significance, but the number of participants was smaller. Some studies have indicated that TL and mtDNA-CN are positively correlated in healthy individuals and in pregnant females [57,58,59], and when this correlation is lost, it may contribute to the progression of some types of cancer, mainly in older patients [4,60]. Mitochondrial dysfunction and its associated generation of oxidative stress are characteristics of PD [61]. On the other hand, the impact of shrinking telomeres on mitochondrial dysfunction via p53 has previously been proposed as an important axis for aging and/or carcinogenesis [62,63]. Therefore, one possible explanation of our finding is a compensation for the insufficient cellular energy supply due to the mitochondrial dysfunction in untreated PD patients, whereby biogenesis reacts to disease pathology (i.e., increasing mtDNA-CN) to maintain normal mitochondrial function in cells [64,65].

Additional reports have shown no correlation between TL and mtDNA-CN in PD blood and brain tissue, implying that the pathways coregulating TL and mitochondrial biogenesis might be disturbed and could also depend upon frequent mtDNA mutation due to PD, which accumulate over time [22], or that the correlation between TL and mtDNA-CN changes from positive to negative depending on the age of the patients, as it has been documented in chronic obstructive pulmonary disease [66].

Within the patients’ group, the mtDNA-CN and TL were higher in those treated than untreated (T0 vs. T1). These significant longitudinal differences of both markers of aging, exclusively in the group of patients with PD, made us wonder about the influence of each DRT as a factor modulating these changes. The comparisons of T0 vs. T1 in the co-treatment groups showed significant differences for both markers of aging, whereas pramipexole treatment was significantly different only for TL. An investigation did not find any correlation between TL and the dose of DRT in PD patients [17], but another study demonstrated evidence that moderate dopamine dose therapy benefits PD patients via the attenuation of oxidative stress and manipulation of the mtDNA-CN [21]. Later, one report in Swedish PD patients found lower mtDNA-CN and longer TL compared to controls in blood and brain tissues. They also observed that TL positively correlated with the medication (levodopa dose) and disease duration [22]. Neuronal mtDNA-CN has been also found to increase with age in controls, but not in idiopathic PD patients [19]. Possible explanations for the discrepancies observed for aging markers in PD could be the type of tissue studied (neurons vs. PBMCs), comedication and presence of comorbidities in the patients, and the start of dopaminergic treatment, which perhaps has an early effect not documented before and our longitudinal design did allow us to observe. A plethora of factors modulates both aging markers; thus, we believe that longitudinal studies as the present one, but including as many variables as possible, may help to determine in a more precise way whether TL and/or mtDNA-CN are markers of progression of PD or are associated with specific characteristics of the disease.

Our preliminary results suggest that erosion of TL and changes in mtDNA-CN could be related to inflammation, and that dopamine agonists/precursors treatment might influence both markers in PD patients. Further studies should confirm these findings. For instance, in 2021, one GWAS reported significant single-nucleotide variants grouped into three clusters that represent distinct functional domains related to mtDNA-CN. These groups included platelet activation, megakaryocyte proliferation, and mtDNA metabolism, which deserve to be thoroughly investigated [23]. Mitochondrial dysfunction and defective autophagy are hallmarks of PD. Inflammation and mitochondria-specific autoimmunity are emerging as important components of neurodegeneration, highlighting the interplay between the immune and nervous systems [67].

A significant correlation between mtDNA-CN and IL-17A plasma levels in untreated PD patients (r = 0.45, *p* < 0.05 at T0) was identified. Plasma levels of IL-17A were previously documented as lower in these patients than in controls (*p* = 0.014) [36]. On the contrary, the present findings suggest an inflammatory profile only in untreated PD patients associated with increased peripheral mtDNA-CN and IL-17A levels. A previous study reported mtDNA changes in blood and urine potentially related to a specific inflammatory response, including IL-17A serum levels in kidney disease in type 2 diabetes mellitus patients [68]. IL-17A is a proinflammatory cytokine that exerts pleiotropic functions activating other inflammatory cytokines. IL-17A is also an important bridge between inflammation and immunity, and it can induce mitochondrial dysfunction by stimulating intracellular ROS production, thereby promoting pyroptosis, as shown in a colorectal cancer study [69]. Elevated mtDNA-CN means mitochondrial dysfunction which leads to oxidative stress and increases the production of proinflammatory cytokines. It would be worthy to further study this longitudinally in larger samples.

Some of the limitations of our work reside in the small size of the sample analyzed and the lack of diverse clinical data in the 2 year follow-up, which did not allow us to perform more detailed analysis with a biological context of our data. However, it is the first time that markers of aging and inflammation in a Latin American population have been analyzed simultaneously and longitudinally. For this reason, we believe that the sample studied is valuable and provides relevant information that deserves further research. Additional studies could explore how changes in TL, mtDNA-CN, inflammation, and other aging markers relate to neurodegeneration in PD.

## 5. Conclusions

To the best of our knowledge, this is the first study to demonstrate longitudinal alterations in mtDNA-CN and TL in PD patients before and after receiving DRT. Our results suggest that aging-associated molecular mechanisms may contribute to the pathophysiology of PD and the response to DRT, with potential opportunities for future therapeutic interventions.

## Figures and Tables

**Table 1:** Demographic characteristics of patients with Parkinson’s disease and healthy controls.

Characteristics	Patients (*n* = 27)			Controls (*n* = 22)		
	Total (*n* = 27)	Male (*n* = 15)	Female (*n* = 12)	Total (*n* = 22)	Male (*n* = 12)	Female (*n* = 10)
Demographic data						
Sex (M%, F%)	100	56	44	100	55	45
Age in years mean ± SD (range)	61.17 ± 10.68 (41–85)	61.73 ± 9.60 (47–85)	63.00 ± 10.82 (41–81)	55.73 ± 10.20 (34–81)	54.58 ± 8.39 (40–69)	57.00 ± 12.38 (34–81)
BMI	27.77 ± 4.78	27.71 ± 3.50	28.02 ± 6.36	26.85 ± 3.89	26.13 ± 3.21	27.72 ± 4.60
Treatment status						
Dopamine agonists (pramipexole)	19	11	8	NA	NA	NA
Dopamine precursors (levodopa)	3	2	1	NA	NA	NA
Agonist + precursor of dopamine	5	2	3	NA	NA	NA
T0	27	15	12	22	12	10
T1	21	12	9	18	10	8
T2	9	4	5	12	7	5
Immunological parameters						
TNF-α (pg/mL)	19.32 ± 17.86	22.93 ± 18.60	14.81 ± 16.54	26.17 ± 29.64	34.15 ± 33.20	16.59 ± 22.71
GM-CSF (pg/mL)	34.69 ± 18.54	34.25 ± 20.35	35.27 ± 16.95	38.08 ± 44.51	30.90 ± 14.04	46.70 ± 65.05
TGF-β (pg/mL)	4452.11 ± 630.46	4427.39 ± 202.12	4483.01 ± 941.07	4349.04 ± 563.05	4361.67 ± 717.53	4336.41 ± 392.52
IFN γ (pg/mL)	6.28 ± 7.50	5.33 ± 6.22	7.47 ± 9.00	4.07 ± 3.34	4.39 ± 4.24	3.69 ± 1.93
IL-10a (pg/mL)	3.09 ± 2.09	2.58 ± 1.95	3.71 ± 2.18	2.69 ± 2.54	3.12 ± 3.27	2.17 ± 1.20
IL-17a (pg/mL)	1273.40 ± 1349.54	1245.07 ± 1407.68	1308.82 ± 1334.30	2250.72 ± 1365.93	2354.57 ± 1370.28	2126.10 ± 1423.70
Neutrophil/lymphocyte inflammation index (NLI)	1.68 ± 0.48	1.72 ± 0.53	1.63 ± 0.41	1.99 ± 1.56	1.81 ± 0.58	2.26 ± 2.22
Systemic Inflammatory index (SII)	398.75 ± 185.11	384.55 ± 166.88	418.11 ± 214.39	492.84 ± 454.88	415.91 ± 186.73	554.47 ± 688.78
Clinical scales for the staging of the functional disability of patients and depression						
HOEN and YAHR scale (median value and range)	2 (1–4)	2 (1–3)	2 (1–4)	NA	NA	NA
MDS-UPDRS I: Non-Motor Experiences of Daily Living (median value and range)	2.5 (0–5)	3 (0–5)	2 (0–4)	NA	NA	NA
MDS-UPDRS II: Motor Experiences of Daily Living (mean value ± SD)	12.30 (±5.90)	12.20 (±6.17)	12.45 (±5.80)	NA	NA	NA
MDS-UPDRS III: Motor Examination (mean value ± SD)	32.69 (±16.53)	31.06 (±14.37)	34.90 (±19.60)	NA	NA	NA
MDS-UPDRS IV: Motor Complications *	NA	NA	NA	NA	NA	NA
MDS-UPDRS Total (mean value ±SD)	47.53 (±22.38)	46.13 (±21.14)	49.45 (±24.88)	NA	NA	NA
Schwab and England (Activities of Daily Living) scale (median value and range)	90 (20–100)	90 (50–100)	80 (20–90)	100 (90–100)	100 (100–100)	100 (90–100)
Beck’s Depression Inventory (median value and range)	10 (0–30)	10 (0–23)	10 (2–30)	2.5 (0–21)	1.5 (0–13)	7 (0–21)

BMI, body mass index, was calculated using a participant’s height and weight. NA, not applicable. * Patients were naive to dopaminergic treatment at this time point of evaluation (T0).

## Data Availability

The data presented in this study are available upon request from the corresponding author. The data are not publicly available due to ethical issues.

## Supplementary Materials

The following supporting information can be downloaded at: https://www.mdpi.com/article/10.3390/genes14101913/s1, Table S1: Comparisons of demographic characteristics and immunological parameters between patients with Parkinson’s disease and healthy volunteers. Table S2: Comparison of mtDNA copy number (mtDNA-CN) and telomere length (TL) in patients (*n* = 27) and controls (*n* = 22). Table S3: Longitudinal comparisons of mitochondrial DNA copy number and telomere length according to the dopaminergic replacement therapy received in PD patients.
