# Supplementary material for: Longitudinal Changes in Mitochondrial DNA Copy Number and Telomere Length in Patients with Parkinson’s Disease
Source: Genes (Basel). 2023 Oct 7;14(10):1913. doi: 10.3390/genes14101913 (PMC10606744; doi:10.3390/genes14101913)
Supplement: Supplementary file 1 [file genes-14-01913-s001.zip › Table S1. Comparisons of demographic characteristics and immunological parameters between patients with Parkinson disease and healthy volunteers..pdf]

Table S1. Comparisons of demographic characteristics and immunological parameters between patients with Parkinson's disease and healthy volunteers.

**Characteristics**	**Patients (*n*=27)**				**Controls (*n*=22)**						
	**Total (*n*=27)**	**Male (*n*=15)**	**Female (*n*=12)**	***P* value**	**Total (*n*=22)**	**Male (*n*=12)**	**Female (*n*=10)**	***P* value**	**Total *P* value**	**Male *P* value**	**Female *P* value**
Demographic data											
**Sex (M%, F%)**	100	56	44	NA	100	55	45	NA	NA	NA	NA
**Age in years mean±SD (range)**	61.17±10.68 (41-85)	61.73±9.60 (47-85)	63.00±10.82 (41-81)	0.2844	55.73±10.20 (34-81)	54.58±8.39 (40-69)	57.00±12.38 (34-81)	0.4200	**0.0486**	0.1820	0.2439
**BMI**	27.77±4.78	27.71±3.50	28.02±6.36	0.6094	26.85±3.89	26.13±3.21	27.72±4.60	0.8052	0.3010	0.4619	0.5161
**Immunological parameters**											
**TNF-alpha (pg/mL)**	19.32±17.86	22.93±18.60	14.81±16.54	0.3076	26.17±29.64	34.15±33.20	16.59±22.71	0.1728	0.2667	0.1388	0.7516
**GM-CSF (pg/mL)**	34.69±18.54	34.25±20.35	35.27±16.95	0.7251	38.08±44.51	30.90±14.04	46.70±65.05	0.4134	0.6582	0.7780	0.5956
**TGF-beta (pg/mL)**	4452.11±630.46	4427.39±202.12	4483.01±941.07	0.9375	4349.04±563.05	4361.67±717.53	4336.41±392.52	0.9731	0.4099	0.4024	0.6340
**IFN gamma (pg/mL)**	6.28±7.50	5.33±6.22	7.47±9.00	0.4357	4.07±3.34	4.39±4.24	3.69±1.93	0.5892	0.1858	0.5855	0.1784
**IL-10a (pg/mL)**	3.09±2.09	2.58±1.95	3.71±2.18	0.0865	2.69±2.54	3.12±3.27	2.17±1.20	0.4013	0.5260	0.5485	**0.0430**
**IL-17a (pg/mL)**	1273.40±1349.54	1245.07±1407.68	1308.82±1334.30	0.9769	2250.72±1365.93	2354.57±1370.28	2126.10±1423.70	0.6544	**0.0157**	**0.0292**	0.1358
**NLI**	1.68±0.48	1.72±0.53	1.63±0.41	0.7345	1.99±1.56	1.81±0.58	2.26±2.22	0.5333	0.2901	0.4941	0.3849
**SII**	398.75±185.11	384.55±166.88	418.11±214.39	0.7160	492.84±454.88	415.91±186.73	554.47±688.78	0.4089	0.2825	0.4724	0.3872

NLI, Neutrophil/lymphocyte ratio; SII, Systemic Inflammatory index; BMI, body mass index was calculated using a participant's height and weight. NA, not applicable. *Patients were naive to dopaminergic treatment at this time point of evaluation (T0). The significant p-values are in bold (total P value means for the complete sample, while the other p-values were calculated stratifying by sex). The t-test was used for the comparison of demographic characteristics and immunological parameters between patients with Parkinson's disease and healthy volunteers.
