# Supplementary material for: Longitudinal Changes in Mitochondrial DNA Copy Number and Telomere Length in Patients with Parkinson’s Disease
Source: Genes (Basel). 2023 Oct 7;14(10):1913. doi: 10.3390/genes14101913 (PMC10606744; doi:10.3390/genes14101913)
Supplement: Supplementary file 1 [file genes-14-01913-s001.zip › Table S2.Comparison of mtDNA-CN and TL in patients and controls.pdf]

Table S2. Comparison of mtDNA copy number (mtDNA-CN) and telomere length (TL) in patients (n=27) and controls (n=22).

Aging marker												
	**Mean value for relative quantification of mtDNA copy number (mtDNA / nDNA ratio)**						**Mean value for relative quantification of Telomere Length (T/S ratio)**					
**Time point**	**Patients**	**Controls**	**p-value**	**t**	**df**	**$CI_{95\%}$**	**Patients**	**Controls**	**p-value**	**t**	**df**	**$CI_{95\%}$**
**T0**	0.71±0.65	0.79±0.68	0.69	0.40	44.11	-0.31-0.47	0.53±0.24	0.90±0.36	**<0.001***	3.96	32.25	0.18-0.56
**T1**	1.52±1.14	0.95±0.91	0.09	-1.72	35.52	-1.25-0.10	0.91±0.28	1.10±0.17	**0.02**	2.56	33.61	0.04-0.34
**T2**	2.24±1.76	1.40±1.00	0.23	-1.28	11.79	-2.27-0.59	0.59±0.27	1.09±0.18	**<0.001****	4.54	11.68	0.26-0.73
**p-value of the comparison of mtDNA copy number and telomere length in patients and controls at the three time points**												
	**Patients**	**Controls**					**Patients**	**Controls**				
**T0 vs T1**	**<0.001**	0.54					**<0.001**	**0.04**				
**T0 vs T2**	**<0.001**	0.07					0.54	0.07				
**T1 vs T2**	0.19	0.20					0.013	0.86				

***** *p*-value = 0.0003859, ***p*-value = 0.0007254, significant p-values are shown in bold. df, degrees of freedom. CI, confidence interval**.** ANOVA test was used for the comparison of mtDNA copy number (mt DNA-CN) and telomere length (TL) in patients and controls.
