# Supplementary material for: Longitudinal Changes in Mitochondrial DNA Copy Number and Telomere Length in Patients with Parkinson’s Disease
Source: Genes (Basel). 2023 Oct 7;14(10):1913. doi: 10.3390/genes14101913 (PMC10606744; doi:10.3390/genes14101913)
Supplement: Supplementary file 1 [file genes-14-01913-s001.zip › Table S3. P-values for dopamine treatment status and mtDNA copy number (mt DNA-CN) and telomere length.pdf]

Table S3. Longitudinal comparisons of mitochondrial DNA copy number and telomere length according to the dopaminergic replacement therapy received in PD patients.

	Longitudinal comparisons of mtDNA copy number and telomere length according to the dopaminergic treatment received in PD patients					
	**mtDNA-CN**			**TL**		
	**Pramipexole**	**Levodopa**	**Cotreatment**	**Pramipexole**	**Levodopa**	**Co-treatment**
Model p-value	0.2314†	0.17†	**0.0044**†	**0.0044**†	0.2030‡	**0.0136**‡
Time treatment status						
**T-0 vs T-1**	0.65	0.45	**0.0017**	**0.0017**	0.3682	**0.0111**
**T-1 vs T-2**	1.0	0.82	0.3786	0.3786	0.2405	0.1738
**T-2 vs T-0**	0.65	0.45	0.9577	0.9577	0.8826	0.2010

†Kruskal-Wallis and ‡ANOVA tests. Statistically significant values are in bold. Co-treatment= levodopa + pramipexole.
